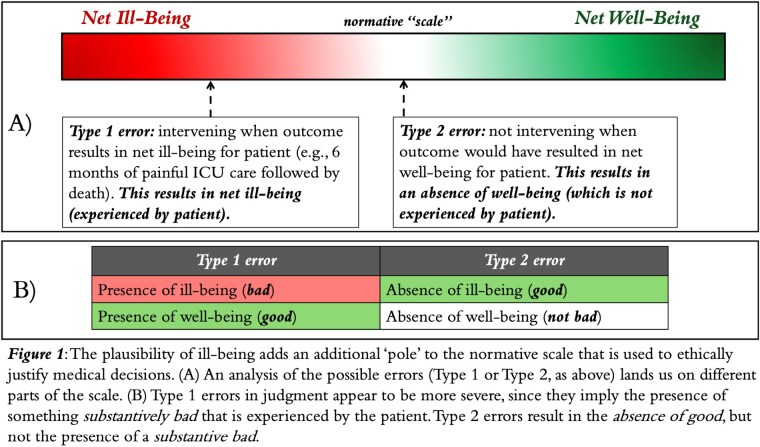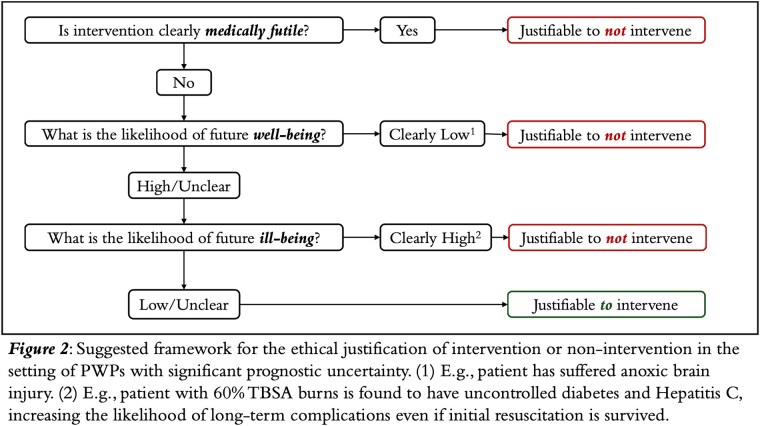# 126 Ill-Being and its Relevance to Unrepresented Burn Patients

**DOI:** 10.1093/jbcr/iraf019.126

**Published:** 2025-04-01

**Authors:** Karel-Bart Celie, Eloise Stanton, Maxwell Johnson, Cindy Rutter, Justin Gillenwater, Haig Yenikomshian

**Affiliations:** University of California Keck School of Medicine; University of California Keck School of Medicine; University of California Keck School of Medicine; Southern California Burn Model System; Los Angeles General Medical Center; University of California Keck School of Medicine

## Abstract

**Introduction:**

Patients-without-proxies (PWPs) lack decision-making capacity, a surrogate, and an advanced directive. Their values are therefore unknown. Due to an aging population and a nation-wide homelessness crisis, PWPs represent a large and growing proportion of patients (Kim and Song, 2018), including those in ICUs (White et al., 2006). Our burn unit routinely cares for critically ill PWPs. Many of these individuals are homeless: nearly half of all individuals in the U.S. who live in a state of chronic homelessness reside in California. Previous work has shown that PWPs are up to 5 times less likely to receive palliative care despite eventual death compared to patients with surrogates (Ota et al., 2020: 10). This exposure of an already vulnerable group to unnecessary suffering suggests there is “a need for a cultural shift in burn care to prevent the suffering of these marginalized patients.”

PWPs in burn care often arrive in critical condition. The current paradigm for medical decision-making when there is prognostic uncertainty focuses exclusively on future well-being. Harm is thus conceived as a lack of well-being. However, we posit that at least some of the suffering that burn patients experience represents not just a lack of well-being, but a substantive/intrinsic harm. Such substantive harm has been termed ‘ill-being’ (Kagan, 2014). Ill-being is thus a morally relevant phenomenon that is almost never accounted for. The purpose of this paper is to present a framework that incorporates ill-being into the ethical justification of intervention or non-intervention in PWPs whose medical prognosis is uncertain.

**Methods:**

The concept of ill-being was incorporated into an ethical analysis of the aforementioned scenarios.

**Results:**

Our analysis demonstrates that, when taking ill-being into account, there is a clear asymmetry of the kinds of errors in judgment that can be made (Figure 1). We propose a framework for ethical justification of intervention vs. non-intervention that incorporates ill-being (Figure 2).

**Conclusions:**

Ill-being is a plausible, morally-relevant concept in the setting of burn care that has been severely neglected. Our analysis shows that the kinds of errors related to the intervention decision are not equal. Type 1 errors are more severe, which warrants an explicit incorporation of ill-being into decision-making. Our proposed framework provides a pathway for doing this.

**Applicability of Research to Practice:**

Research on PWPs is in a “dismal state” and there is an “urgent need for research to identify medical decision-making approaches across health-care settings” (Kim and Song, 2018: 1233). The suggested framework is one such approach.

**Funding for the Study:**

N/A